# The Effect of Post-Processes on the Microstructure and Creep Properties of Alloy718 Built Up by Selective Laser Melting

**DOI:** 10.3390/ma11060996

**Published:** 2018-06-12

**Authors:** Yen-Ling Kuo, Toshiki Nagahari, Koji Kakehi

**Affiliations:** Department of Mechanical Systems Engineering, Tokyo Metropolitan University, 1-1 Minami-osawa, Hachioji-shi, Tokyo 192-0397, Japan; nagahari-toshiki@ed.tmu.ac.jp (T.N.); kkakehi@tmu.ac.jp (K.K.)

**Keywords:** superalloy, Alloy718, selective laser melting, post-process, heat treatment, hot isostatic pressing

## Abstract

The selective laser melting (SLM) process was used to fabricate an Alloy718 specimen. The microstructure and creep properties were characterized in both the as-built and post-processed SLM materials. Post-processing involved several heat treatments and a combination of hot isostatic pressing (HIP) and solution treatment and aging (STA) to homogenize the microstructure. The experimental results showed that the originally recommended heat treatment process, STA-980 °C, for cast and wrought materials was not effective for SLM-processed specimens. Obvious grain growth structures were obtained in the STA-1180 °C/1 h and STA-1180 °C/4 h specimens. However, the grain size was uneven since heavy distortion or high-density dislocation formed during the SLM process, which would be harmful for the mechanical properties of SLM-fabricated materials. The HIP+ direct aging process was the most effective method among the post-processes to improve the creep behavior at 650 °C. The creep rupture life of the HIP+ direct aging condition approached 800 h since the HIP process had the benefit of being free of pores, thus preventing microcrack nucleation and the formation of a serrated grain boundary.

## 1. Introduction

The IN718 superalloy is widely used in gas turbine and related aerospace applications due to its excellent mechanical properties and structural stability at elevated temperatures [1,2,3]. However, IN718 components produced by conventional processing techniques are limited in terms of their potential complexity and, thus, in their operating range and efficiency [4]. Additive manufacturing processes such as selective laser melting (SLM) offer several advantages in comparison to conventional processing techniques, such as a large degree of design flexibility and reductions in the number of production steps, lead time, and investment cost [5,6,7,8]. Additive manufacturing fabrication has become an increasingly attractive fabrication method due to the above-mentioned advantages over conventional manufacturing methods. However, the strong thermal gradient, which is induced by the highly localized heat input, the energy absorption, and the rapid solidification rate, results in promotion of residual stress during the SLM process. Moreover, the fast solidification rate is also responsible for the microsegregation of the chemical composition and the formation of nonequilibrium phases, which are associated with the formation of dendritic growth [9,10]. As shown in [9], Amato et al. indicated that post-treatments may promote the formation of secondary precipitates, namely, Laves and carbides, which will affect the mechanical properties of the Inconel625 superalloy built up by SLM. On the other hand, in our previous experience with the solution treatment and aging (STA) process (980 °C/1 h), which is the originally recommended treatment for conventional Alloy718, the creep behavior of Alloy718 built up by SLM showed poor creep rupture life [11]. The row of interdendritic δ precipitates with incoherent interfaces acted as a nucleation site for damage accumulation and led to a decrease in creep life. Moreover, the existence of residual stress that was blocked by the subgrain boundary prohibited dislocation motions and accelerated the rate of crack propagation. Therefore, a suitable post-process is required to improve the microstructure and mechanical properties of SLM materials. The normal post-processes recommended for additive-manufactured Ni-based superalloys are thermal treatments and hot isostatic pressing (HIP) [12,13,14]. Due to the metastable property of the primary strengthening phase (γ″) in Alloy718 and the ability for undesirable phases to form, determining optimal post-processes for Alloy718 can be difficult [15,16]. The current work is seen as a logical continuation of our previous work on the effect of different post-processes on the microstructure, texture, and creep properties of Alloy718 superalloy built up by SLM. In this study, several heat treatment (HT) processes and HIP were conducted to improve the microstructure and mechanical properties of SLM materials. The objective of this study is to analyze the effects of post-processes on the microstructure and creep properties of Alloy718 built up by the SLM process.

## 2. Materials and Experimental Procedure

The chemical composition of the Alloy718 powder is presented in Table 1. A set of SLM process parameters for Alloy718 provided by GmbH Electro Optical Systems (EOS) (Robert-Stirling-Ring 1, 82152, Krailling, Bavaria, Germany) (laser power 400 W; scanning speed 7.0 m/s; hatch distance 80 μm; layer thickness 40 μm; beam diameter 100 μm; atmosphere pure 99.9999% argon) was utilized to fabricate an Alloy718 cube with the dimensions 35 mm × 35 mm × 35 mm. A multidirectional scanning strategy was used in which the laser scan direction was rotated by 66.5° for each layer to reduce the residual stress. After the SLM process, test specimens were cut from the cube parallel to the build direction using a spark cutter, and the dimensions of each were 19.6 mm × 2.8 mm × 3.0 mm. Each specimen was then divided into several sets of samples for different post-processes. Table 2 summarizes the post-processes for the SLM samples. For Alloy718, solid solution and precipitation strengthening are the major steps in the strengthening mechanisms. Based on the strengthening mechanisms, the heat treatment scheme consisted of solution treatment and aging (STA) [17]. In this work, the as-built specimen was heated to various solution temperatures ranging from 980 to 1180 °C (Table 2), kept at each temperature for 1 h, and allowed to cool to room temperature by air cooling. Subsequently, each solution-treated specimen was given a two-step aging treatment consisting of 718 °C for 8 h, furnace cooling to 621 °C, holding at 621 °C for 10 h, and air cooling to room temperature. These heat-treated samples were designated STA-980 °C, STA-1045 °C, STA-1065 °C, STA-1120 °C, and STA-1180 °C (Table 2). On the other hand, some as-built samples were directly subjected to an optimal condition for HIP at 1180 °C and 175 MPa for 4 h (abbreviated as as-HIPed) [18]. One more condition was studied in this research, namely, HIP+ direct aging, which involved undergoing an aging treatment after the HIP process. After post-processes, the tensile creep test was carried out at 650 °C and 550 MPa. The microstructures were observed using a scanning electron microscope (HITACHI S-3700N; Hitachi, Tokyo, Japan) and a transmission electron microscope (TEM) (JEOL JEM-3200FS; JEOL, Tokyo, Japan). Inverse pole figures (IPF) and Kernel Average Misorientations (KAM) maps were calculated from the orientation measurements by electron backscatter diffraction (EBSD) (2.2 SP2, Oxford Instruments, Oxfordshire, UK).

### Design of Post-Processes

The purpose of post-processes is to obtain an isotropic and homogenous microstructure to realize high creep resistance and ductility. In this work, as-built specimens were heated to desired solution temperatures in the range from 980 °C to 1180 °C (summarized in Table 2), kept at the temperatures for 1 h, and allowed to cool to room temperature by air cooling. After the solution treatment, the specimens were subjected to a two-step aging treatment consisting of 718 °C for 8 h, furnace cooling to 621 °C, holding at 621 °C for 10 h, and air cooling to room temperature (Table 2). The purpose of heat treatment is to dissolve segregation particles and strengthening phases into the matrix and to re-precipitate the γ′ and γ″ phases with the following double aging treatment. This treatment avoids the poor mechanical characteristics of Alloy718 built up by SLM in our previous study [2]—i.e., the high dislocation density, the subgrain boundary, and the row of undesired precipitates.

In this study, solutions were treated at the subsolvus (980 °C), solvus (1045 °C), and supersolvus (1065 °C) temperature of the undesired δ phase to evaluate the effect of the δ phase on the creep properties. On the other hand, treatment at 1100 °C rather than 980 °C has been suggested to achieve sufficient delta dissolution for traditionally processed Alloy718 [18]. Moreover, solutions treated at 1100 °C have sufficient energy at the beginning of grain growth, which is associated with a release of the stored strain energy through the annihilation of dislocations [19]. Further, coarsening of the grain structure was observed with heating above 1100 °C in a laser-solid-formed superalloy. Therefore, solution treatments at 1100 °C, 1120 °C, and 1180 °C were compared in this study. After the solution treatment, the specimens were subjected to a two-step aging treatment as mentioned above.

After completion of the build, some as-built specimens were subjected to the HIP process, which could eliminate the small amount of residual porosity and segregation to obtain a homogenous microstructure [12,13]. An optimal HIP condition for traditionally processed Alloy718, consisting of a soaking temperature of 1180 °C and a pressure of 175 MPa for 4 h, was used [17]. After the HIP process, the as-HIPed specimens were subjected to a double aging process. Moreover, an STA-1180 °C/4 h specimen was solution-treated at 1180 °C and kept at the temperature for 4 h, followed by furnace cooling to room temperature (an equivalent temperature and time to HIP process) and subsequently subjected to double aging, then compared to the other variants in order to investigate the effect of pressure in the HIP process on the mechanical properties of SLM materials in this study. Comparisons of density between post-processed materials and cast and wrought alloys using the Archimedes method are shown in Table 3.

## 3. Results

### 3.1. Microstructure Changes

#### 3.1.1. The As-Built Microstructure

**Figure 1:**
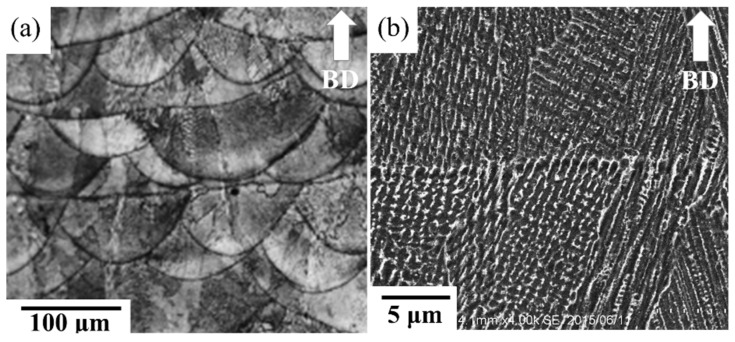
Secondary electron images of the as-built specimen: (**a**) lower magnification showing the molten pools structure and (**b**) higher magnification showing dendritic structures. The build direction with respect to the plane of the images is shown with an arrow.

**Figure 2:**
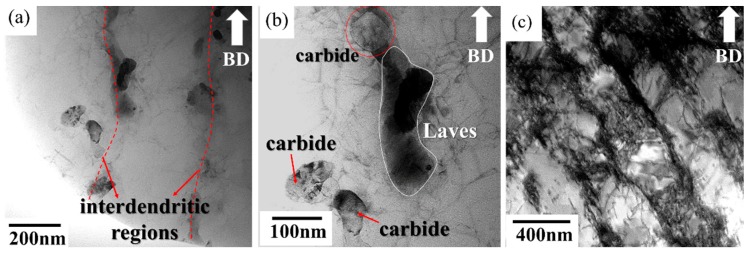
TEM images of as-built specimen showing (**a**) precipitates along the interdendritic region, (**b**) interdendritic precipitates, and (**c**) high-density dislocation. The build direction with respect to the plane of the images is shown with an arrow.

Figure 1 shows the microstructure of the as-built specimen. In the side view, molten pool boundaries were observed in the typical arc-shaped configuration; they were 75 μm in thickness and 100 μm in width and were induced by the Gaussian energy distribution of the laser [20]. In addition, the dendrite structure and interdendritic regions were decorated with a continuous network of precipitates which were identified as Laves phase and carbides by TEM analysis (Figure 2). The terminal stages of solidification consist of a primary L → γ stage followed by L → γ + NbC and L → γ + Laves reactions due to the segregation of Nb (Figure 2) [21]. In addition, high-density dislocations originated from the production of thermal stress during the melt-solidification processing (Figure 2c) [22].

The shaping process (rapid heating and cooling) induces thermal variations that cause areas of the selective-laser-processed layers to expand and contract at different rates, generating high-density dislocations.

**Figure 3:**
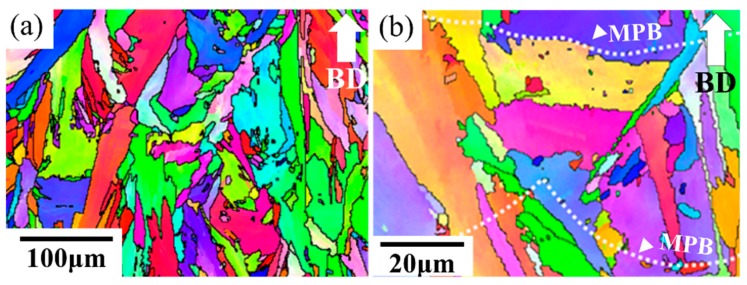
Inverse pole figure (IPF) maps of the as-built specimen were taken at (**a**) low magnification and (**b**) high magnification. The build direction with respect to the plane of the images is shown with an arrow.

The IPF map of the as-built specimen was constructed by means of EBSD as shown in Figure 3. A mixture of columnar grains and equiaxed grains was observed from the side view (Figure 3). The microstructure can be attributed to the heat flux during the SLM solidification process [4]. The equiaxed grains generally resulted from the heterogeneous nucleation on the partially melted areas, such as the overlapped areas or the areas near the solidification front [23]. On the other hand, the epitaxial growth led to the columnar grains.

#### 3.1.2. The Heat-Treated Microstructure

**Figure 4:**
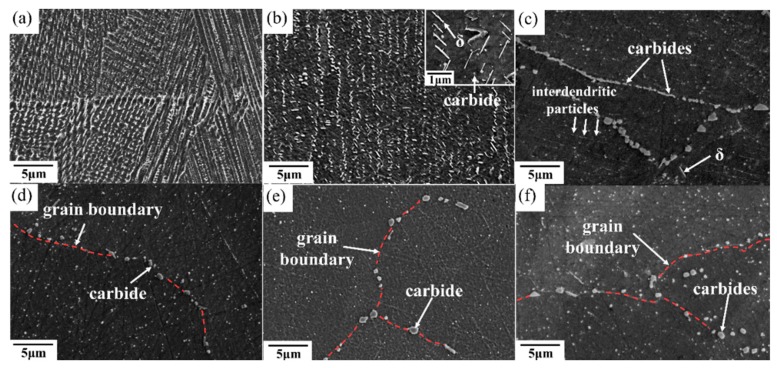
Scanning electron microscope images of (**a**) as-built, (**b**) STA-980 °C, (**c**) STA-1045 °C, (**d**) STA-1065 °C, (**e**) STA-1120 °C, and (**f**) STA-1180 °C/1 h specimens.

**Figure 5:**
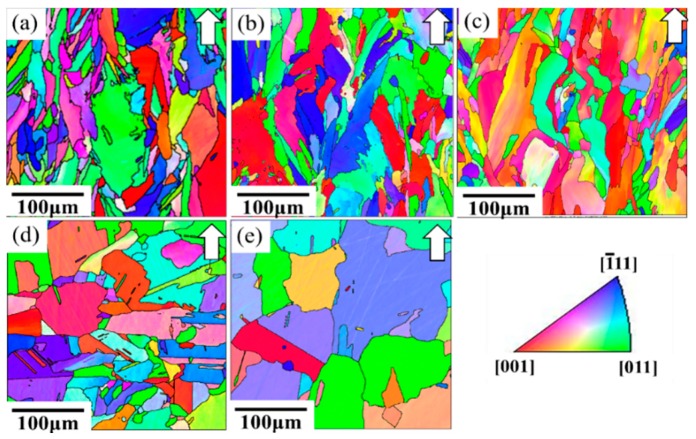
IPFs of (**a**) STA-980 °C, (**b**) STA-1045 °C, (**c**) STA-1065 °C, (**d**) STA-1120 °C, and (**e**) STA-1180 °C/1 h specimens were analyzed using the orientation measurements by EBSD. The building directions are shown by arrows.

After the solution treatment at 980 °C and the double aging heat treatment, there were numerous Nb-rich precipitates and δ phases along the grain boundaries and interdendritic regions (Figure 4b). The transformation of Laves into δ phases is a result of the solution heat treatment under the STA-980 °C condition. Similar results have also been reported in [24]. The chemical compositions of Laves in the as-built specimen and that of the δ phase under the STA-980 °C condition are shown in Table 4. This needle-shaped δ phase is generally undesirable due to its adverse effect on the mechanical properties. Further, the pinning effect of the δ phases resulted in similar grain morphology and grain size between the STA-980 °C specimen and the as-built specimen (Figure 5). This standard solution treatment at 980 °C is not effective for the homogenization of SLM-processed specimens. Figure 4c,d show the microstructures that went through the STA-1045 °C and STA-1065 °C conditions, respectively. Continuous carbides were distributed along the grain boundaries in the STA-1045 °C specimen, which showed spherical carbides along the grain boundaries. Moreover, the amount of the interdendritic δ phase decreased as the solution temperature increased. In addition, as shown in Figure 5c, a solution temperature of 1065 °C—above the temperature of the delta solvus (1045 °C)—led to slight grain growth due to elimination of the δ phase, which inhibits recrystallization and grain growth control through the pinning of grain boundaries.

**Figure 6:**
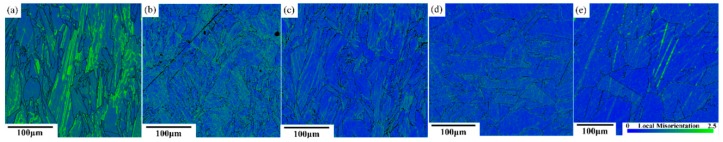
KAM maps of (**a**) as-built, (**b**) STA-1045 °C, (**c**) STA-1065 °C, (**d**) STA-1120 °C, and (**e**) STA-1180 °C/1 h specimens were analyzed using the orientation measurements by EBSD. A square scanning grid and neighbor shell (5 × 5) were used to calculate the misorientation. These KAM maps were run at a 0.5 μm step size using a 600 × 600 point grid.

After STA-1120 °C and STA-1180 °C, most of the δ phase had been dissolved back into the matrix, as shown in Figure 4e,f. However, the carbides consisting primarily of NbC became coarse at temperatures above 1120 °C, since the material had sufficient time for carbides to grow. Moreover, the driving force for carbide precipitation is the segregation of the carbide-forming elements—e.g. carbon and niobium—as the alloy cooled [25]. It was also noted that grain growth became active during the solution treatment at 1120 °C (Figure 5d). Kernel Average Misorientations (KAM) maps show an obvious decrease in the dislocation density in the STA-1120 °C specimen (Figure 6).

However, the grain size is uneven in the favored sites because the driving force for grain growth can often be characterized as regions of heavy distortion or high dislocation density [26], like the overlapping areas in SLM materials.

#### 3.1.3. The HIPed Microstructure

**Figure 7:**
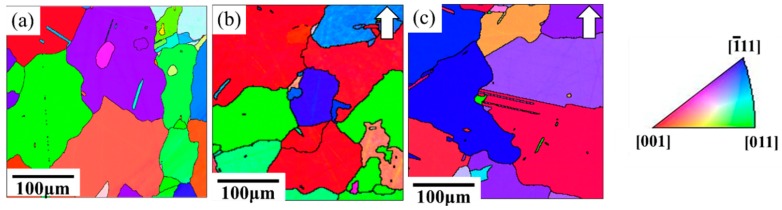
IPF images of (**a**) STA-1180 °C/4 h, (**b**) as-HIPed, and (**c**) HIP+ direct aging specimens were analyzed using the orientation measurements by EBSD. The build directions are shown by arrows.

**Figure 8:**
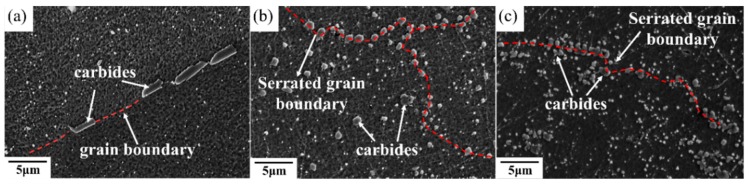
Scanning electron microscope images of (**a**) STA-1180 °C/4 h, (**b**) as-HIPed, and (**c**) HIP+ direct aging specimens.

An equiaxed-grains structure was observed after the HIP process (Figure 7b,c). The high HIP temperature led to great grain growth and a more isotropic appearance with coarsening grains compared to heat-treated SLM specimens. Figure 7 shows the IPF maps of specimens that went through the HIP process and direct aging treatment (abbreviated HIP+ direct aging). As can be observed, the subsequent heat treatment resulted in annealing twins. Moreover, the HIPed materials exhibited a much greater volume fraction of coarser carbide due to the lower solidification rates during the HIP process (Figure 8). The increase in carbide size is consistent with the increase in the time available for growth at the lower cooling rates encountered in HIP processing. The carbides were densely distributed along the grain boundary and also uniformly in the matrix in HIPed specimens, while the STA-1180 °C/4 h specimen showed that carbides mostly precipitated along the grain boundaries (Figure 8).

### 3.2. Mechanical Properties of Heat-Treated SLM Specimens

**Figure 9:**
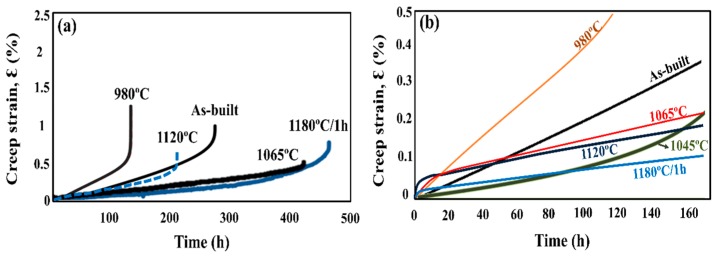
(**a**) Creep curves of heat-treated specimens under 650 °C/550 MPa and (**b**) in the early stage of the initial 165 h.

**Figure 10:**
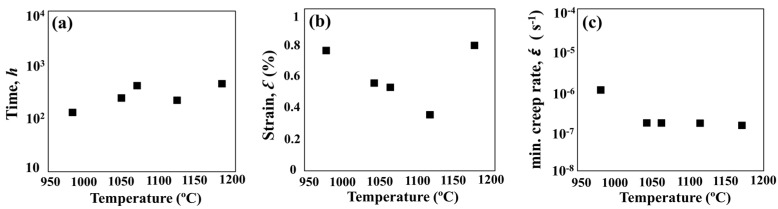
(**a**) Creep life, (**b**) creep rupture elongation, and (**c**) the minimum creep rate of heat-treated specimens under 650 °C and 550 MPa.

**Figure 11:**
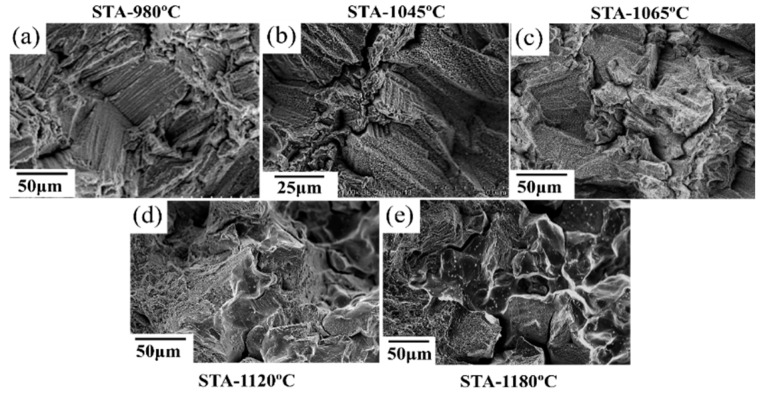
Rupture surfaces of (**a**) STA-980 °C specimen, (**b**) STA-1045 °C specimen, (**c**) STA-1065 °C specimen, (**d**) STA-1120 °C specimen, and (**e**) STA-1180 °C/1 h specimen under 550 MPa at 650 °C.

To study the effect of heat treatment on the mechanical properties, a creep test was performed. All of the heat-treated specimens were subjected to creep under 650 °C/550 MPa. The effects of heat treatment on the creep properties of SLM specimens are shown in Figure 9 and Figure 10. The minimum creep rate was measured in the early portions of the test (see Figure 10c). As shown in Figure 9, the as-built specimen exhibited a creep rupture life of 270 h, while the STA-980 °C specimen exhibited only half the rupture life of the as-built specimen. The presence of successive needle-shaped δ precipitates, in conjunction with the poor coherency, contributed to the inferior creep life and poor elongation in STA specimens. The row of interdendritic δ precipitates with incoherent interfaces acted as nucleation sites for damage accumulation and a decrease in creep life. The δ-phase embrittlement would be the primary reason that the STA-980 °C specimen showed the poorest creep rupture life [11]. The STA-1045 °C condition produced a creep rupture life two times longer than that of the STA-980 °C specimen. As the treatment used a higher solution temperature, the STA-1065 °C specimen showed a much longer creep rupture life compared with the STA-980 °C specimen due to the better dissolution of the interdendritic δ phase (Figure 4). Further analysis of the creep curve data revealed that the STA-1045 °C and STA-1065 °C samples reached minimum creep strain rates of 1.59 × 10^−7^ s^−1^ and 1.90 × 10^−7^ s^−1^, respectively (Table 5); this indicates that both specimens had similar creep rates during the early stages of creep, while the STA-1045 °C specimen showed a dominant accelerating creep stage after 100 h (Figure 9). On the other hand, the STA-1065 °C specimen showed a rupture life 1.5 times longer than that of the STA-1045 °C specimen due to the smaller amounts of Laves phase and δ phase. The rupture surfaces showed a mixture of dendritic pattern and transgranular pattern in the STA-980 °C, STA-1045 °C, and STA-1065 °C specimens, as shown in Figure 11a–c, respectively. The presence of the interdendritic δ phase, in conjunction with the poor coherency in the above specimens, brought about the poor creep life. Moreover, Alloy718 is precipitation-strengthened primarily by γ″ phases, which are based on a Ni_3_Nb composition. However, the formation of δ phase requires a niobium concentration ranging from 10% to 30%, which depletes the matrix of the principal alloy elements required for hardening [18]. As a result, the creep strength would decrease as the formation of δ phase increased. Furthermore, the rupture mode would become transgranular when the grain aspect ratio is large [27]. The rupture surface is therefore partly dendritic and partly transgranular.

At conditions above STA-1120 °C, the specimen showed a decreased rupture life (Figure 9a), while the STA-1180 °C/1 h specimen showed improved creep rupture life due to the grain growth. Creep fracture at intermediate temperatures (*T*/*T*_m_ = 0.3 to 0.6, 380 °C–760 °C) is often initiated with the nucleation and growth of cavities on grain boundaries. As a result, the STA-1180 °C/1 h specimen would show better creep resistance than STA-1120 °C due to a lower contribution of grain boundary sliding to the overall deformation. The rupture surfaces showed a mixture of intergranular and transgranular patterns in the STA-1120 °C specimens, as shown in Figure 11d. The long grains rupture in a transgranular pattern, while shorter grains may “pull out”, resulting in a rupture which is partially transgranular and partially intergranular [27]. On the other hand, the STA-1180 °C specimen (Figure 11e) showed intergranular fractures, which is common in creep tests since the homologous temperature of the creep test is intermediate, and the grain boundary is always attributed to the damage initiation.

### 3.3. Mechanical Properties of HIPed SLM Specimens

**Figure 12:**
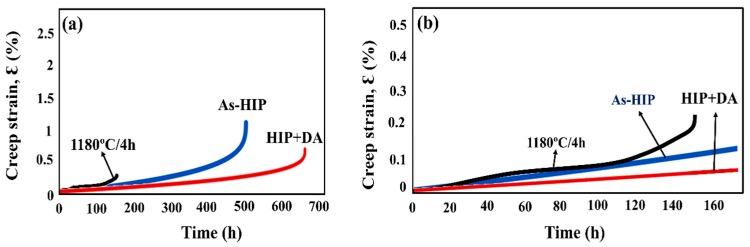
(**a**) Creep curves of post-processed specimens under 650 °C /550 MPa and (**b**) in the early stage of the initial 165 h.

**Figure 13:**
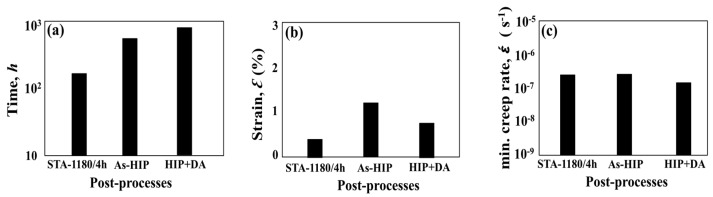
(**a**) Creep life, (**b**) creep rupture elongation, and (**c**) the minimum creep rate of post-processed specimens under 650 °C/550 MPa.

**Figure 14:**
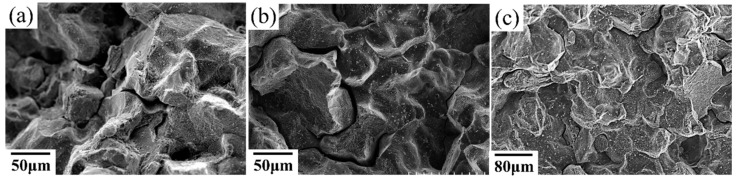
Rupture surfaces of (**a**) 1180/4 h, (**b**) As-HIPed, and (**c**) HIP+ direct aging specimens under 550 MPa at 650 °C.

Although the STA-1180 °C/1 h specimen showed the longest creep rupture life among the heat-treated SLM specimens (Figure 9), the STA-1180 °C/4 h exhibited poor creep properties (Figure 12 and Figure 13). On the other hand, the as-HIPed specimen without heat treatments exhibited a better creep rupture life than the STA-1180 °C/4 h specimen (Figure 9). Moreover, the creep rupture life of the HIP+ direct aging condition approached 700 h, since the principal strengthening phase was precipitated with the subsequent heat treatment. The rupture surfaces of the STA-1180 °C/4 h, as-HIPed, and HIP+ direct aging specimens were observed to be common intergranular fractures (Figure 14).

## 4. Discussion

### 4.1. Effects of the Formation of Laves Phase/δ Phase in SLM Materials

The element Nb has been shown to be very important for controlling the microstructure in Alloy718, which relies on the γ″ (Ni_3_Nb) phase for strengthening [15]. However, Nb is easily segregated into the interdendritic regions during the solidification process. The segregation of Nb leads to the formation of Laves at the end of solidification, which is generally undesirable. Laves is a hexagonally closely packed phase and is generally accepted to be of the form of a high Nb concentration ranging from 10% to 30% [18]. Moreover, the associated formation of the Nb-rich δ phases indicates that much of the Nb will be tied up as secondary phases in the STA-980 °C specimen (Table 4). As a result, the formation of Laves/δ phases would deplete the matrix of principal strengthening elements. Moreover, Laves/δ phases would represent a weak zone between the interfaces of Laves and the matrix, leading to premature fracture. The Laves/δ phases would act as preferential sites for crack initiation and propagation due to their inherent brittle nature [18]. Therefore, the creep strength would decrease as the formation of the Laves phase/δ phase increases along the interdendritic region [21].

### 4.2. Grain Morphologies of the As-Built Specimen

The mixture of columnar grains and equiaxed grains was observed from a side view (Figure 3). These microstructural differences can be attributed to the heat flux during the SLM solidification process [4]. The equiaxed grains generally resulted from the heterogeneous nucleation of the partially melted areas, such as the overlapping areas or the areas near the solidification front [24]. On the other hand, the epitaxial growth led to the development of columnar grains.

### 4.3. Inhomogeneous Grain Growth and Its Effects on Mechanical Properties

Owing to the locally concentrated energy input, repeated rapid heating, fast solidification rate, and consequent plastic deformation, residual stress remained during the SLM process [19], which could be the driving force for the normal grain growth. The grain boundaries with high surface energy serve as the favored locations for grain growth [26]. Active grain growth was observed at the solution temperature of 1120 °C (see Figure 5d). However, the amount of residual stress affects the rate of grain growth because the residual stress alters the surface energy of the grain boundaries. Moreover, the residual stress would affect the inhomogeneity of growth rates [28]. When the sample was further HIP-treated at 1180 °C and 175 MPa for 4 h, an inhomogeneous distribution of fine grains was observed, as shown in Figure 7b,c. This inhomogeneous grain growth led to unevenly distributed grains, which would be harmful for the mechanical properties of additive manufactured materials [29]. Damage initiation is always attributed to the grain boundary, where voids may form and grow by diffusional processes in the creep test when the test temperature is intermediate (*T*/*T*_m_ = 0.3 to 0.6) [26]. A large grain size is preferred for creep resistance, while small regions of equiaxed grains may act as nuclei of failure [26]. The δ phase was dissolved in the matrix by the solution treatment at higher temperatures; however, creep properties were not greatly improved because of the inhomogeneous grain growth.

### 4.4. The Effects of HIP on the Mechanical Properties of SLM Specimens at 650 °C

The experimental results presented in Figure 12 and Figure 13 of the present work suggest that HIP treatment improves the creep behavior. A much longer rupture life and a nearly zero steady-state rate were observed in the HIP+ direct aging specimen. An extremely low steady-state rate would be generally caused by the precipitation process, as the new precipitates would make further dislocation difficult during the creep test [26]. Moreover, a serrated grain boundary with zig-zag morphology was formed in the HIP specimens, which would prolong the creep rupture life significantly [1] (see Figure 8). The serrated boundaries are said to arise from the cellular carbides that are localized at grain boundaries [30]. The zig-zag grain boundaries with carbides can increase the rupture strength by preventing early cavity formation and the linking of growing cavities along grain boundaries [31]. Inhibition of the grain growth by the carbides would bring about the serrated grain boundary. As a result, the HIP process was the most effective among the post-processes (Figure 12), and it has the benefits of being free of pores, inhibiting micro crack nucleation [32], and leading to a serrated grain boundary with a high volume (Figure 8b,c). The present work presents clear experimental evidence for the beneficial effect of HIP on the creep properties.

## 5. Conclusions

In this study, the effects of post-processes on the microstructures and mechanical properties of SLM-fabricated Alloy718 were investigated. The following conclusions can be drawn from this work:

1. The dendrite structure and interdendritic regions were decorated with a continuous network of Laves phase and carbides in the as-built specimen. In addition, the rapid heating and cooling induces thermal variations that cause high-density dislocations.
2. The originally recommended heat treatment process, STA-980 °C, for cast and wrought materials is not effective in SLM-processed specimens.
3. Laves phases/δ phases were dissolved in the matrix by a solution treatment at higher temperatures; however, creep properties were not improved greatly because of the inhomogeneous grain growth.
4. The HIPed materials exhibited a serrated grain boundary with a high-volume fraction of carbide along the grain boundary. HIP improved the creep life, and the HIP+ direct aging process was the most effective among the post-processes for improving the creep behavior at 650 °C.

## Figures and Tables

**Table 1:** Chemical composition of IN718 powder used in selective laser melting (SLM) (mass %).

Cr	Nb	Mo	Ti	Al	Co	Cu	C	Si, Mn	P, S	B	O	Fe	Ni
19.6	5.05	2.85	1.10	0.46	0.03	0.05	0.04	0.04	0.0	0.002	0.019	Balance	52.59

**Table 2:** Post-process variants.

**Variant**	**1st Step: Solution Treatment**	**2nd Step: Age Hardening**
STA-980 °C	980 °C/1 h/air cooling	720 °C/8 h/furnace cooling to 620 °C + 620 °C/10 h/air cooling
STA-1045 °C	1045 °C/1 h/air cooling	
STA-1065 °C	1065 °C/1 h/air cooling	
STA-1120 °C	1120 °C/1 h/air cooling	
STA-1180 °C/1 h	1180 °C/1 h/air cooling	
STA-1180 °C/4 h	1180 °C/4 h/furnace cooling	
**Variant**	**1st Step: HIP Process**	**2nd Step: Post-Treatment**
As-HIPed	HIP at 1180 °C/175 MPa/4 h	N/A
HIP + direct aging	HIP at 1180 °C/175 MPa/4 h	720 °C/8 h/furnace cooling to 620 °C + 620 °C/10 h/air cooling

**Table 3:** Comparisons of density between post-processed materials and cast and wrought alloys.

Variant	Density (g/cm^3^)	Density Compared with Cast and Wrought Alloy (%)
cast and wrought alloy	8.23	100.00
as-built	8.20	99.62
STA-980 °C	8.19	99.46
STA-1045 °C	8.17	99.26
STA-1065 °C	8.20	99.59
STA-1120 °C	8.18	99.38
STA-1180 °C/1 h	8.20	99.61
STA-1180 °C/4 h	8.18	99.32
as-HIPed	8.19	99.45
HIP+ direct aging	8.24	100.11

**Table 4:** TEM-EDS results of the precipitate (atom %).

Elements	Al	Ti	Cr	Fe	Ni	Nb	Mo
Laves (as-built)	2.20	1.09	12.74	11.28	45.42	22.06	5.21
δ phase (STA-980 °C)	0.84	2.43	4.17	3.41	51.16	35.86	2.12
matrix (STA-980 °C)	2.38	0.47	12.45	14.16	61.63	5.59	3.20

**Table 5:** Creep properties of post-processed specimens.

Variant	STA-980 °C	STA-1045 °C	STA-1065 °C	STA-1120 °C	STA-1180 °C/1 h	STA-1180 °C/4 h	As-HIPed	HIP+ Direct Aging
steady-state rate, έ (10^−7^ s^−1^)	10.7	1.59	1.90	1.59	1.37	1.74	1.73	0.89
creep life (h)	134	254	426	230	462	151	493	677
strain (%)	1.29	0.56	0.53	0.75	0.81	0.2	1.10	0.65
